# Preparation, characterization and pharmacokinetics of cyadox nanosuspension

**DOI:** 10.1038/s41598-017-02523-4

**Published:** 2017-05-23

**Authors:** Adeel Sattar, Dongmei Chen, Lishun Jiang, Yuanhu Pan, Yanfei Tao, Lingli Huang, Zhenli Liu, Shuyu Xie, Zonghui Yuan

**Affiliations:** 1 National Reference Laboratory of Veterinary Drug Residues (HZAU), Huazhong Agricultural University, Wuhan, Hubei 430070 China; 2 MAO Key Laboratory for Detection of Veterinary Drug Residues, Huazhong Agricultural University, Wuhan, Hubei 430070 China; 3 MOA Laboratory for Risk Assessment of Quality and Safety of Livestock and Poultry Products, Huazhong Agricultural University, Wuhan, Hubei 430070 China

## Abstract

An increase in number of newly developed synthetic drugs displays bioavailability constraints because of poor water solubility. Nanosuspensions formulation may help to overwhelm these problems by increasing dissolution velocity and saturation solubility. In the present study, cyadox (Cyx) nanosuspension was successfully prepared by recrystallization based on acid–base neutralization combined with high pressure homogenization method using Polyvinylpyrrolidone K30 (PVP) as stabilizer. The nanosuspension had uniform particle distribution, excellent sedimentation rate and redispersibility. The nanosuspension significantly improved the solubility, dissolution and bioavailability. The saturation solubility of Cyx nanocrystal was higher than that of bulk Cyx and released the total drug in very short time. Further, pharmacokinetics of Cyx nanosuspension and normal suspension following oral administration was investigated in beagle dogs. Nanosuspension improved the bioavailability of Cyx which could be beneficial for intestinal bacterial infection in animals. Maximum concentration and area under concentration time curve were increased with particles size reduction which might give rise to pronounce fluctuations in plasma concentration and more intensified antibacterial effects. The terminal half-life and mean resident time of Cyx nanosuspension had also increased compared to normal Cyx suspension. In conclusion, nanosuspensions may be a suitable delivery approach to increase the bioavailability of poorly soluble drugs.

## Introduction

Quinoxaline 1, 4-di-N-oxides (QdNOs) are widely used synthetic antibacterial derivatives in animals to treat many gram-negative and gram-positive bacterial infections^1–3^. The important members of this group including carbadox, olaquindox and mequindox have been strictly limited to use due to potential toxicity in animals. Cyadox (Cyx) is believed to be safer and effective antibacterial agent of QdNOs with much lower toxicity in animals compared to other members of this group^4, 5^. It has been used as an effective antibacterial agent against most of pathogenic bacterial species in animals including Salmonella, Escherichia coli, Pasturella and Staphylococcus species^6, 7^. Dogs are generally considered to more likely to these type of bacterial infections^8, 9^. Considering its exceptional safety as well as efficacy, Cyx might be a promising antibacterial agent to treat infectious diseases in various animal species, including canines.

However, the Cyx has a poor solubility in aqueous medium as well as simultaneously in organic solvents, which is becoming its major challenge in the clinic application for animal infection treatment. The low solubility leads to drug delivery complications like erratic absorption and unsatisfactory oral bioavailability. Due to large requirement of volume, intravenous delivery is also not possible. Currently, there is no effective formulation for Cyx except the premix. Therefore, the enhancement of Cyx solubility should be firstly solved in its development.

Many traditional approaches are adopted to enhance the drug solubility i.e co-solvents, micronization and cyclodextrin. But, bioavailability problem remains as such in many cases, as micronization does not produce a sufficient surface in order to increase the dissolution velocity of poorly soluble drugs. Consequently, the industry has moved forward from micronization to nanonization (production of drug nanocrystals)^10–12^. Production of nanocrystals revolutionized the pharmaceutical industry by improving the performance of poorly soluble drugs. Nanocrystals enhance the saturation solubility as well as dissolution velocity, improved bioavailability, proportionality and oral absorption^13, 14^. Nanocrystals are considered to be a good choice in those conditions where low absorption of the drug is mainly due to dissolution velocity. Not only for oral route of administration, nanosuspension can also be effectively used intravenously as well as other routes like ocular, dermal or pulmonary routes^15^. Because of relatively cheaper cost, larger drug loading capability, production in scale up, and none or fewer carrier-associated side effects, the nanosuspension are easily commercialized^16^. There is currently several related nanocrystal production in human medicine clinic after the first product Emend® introduced in the market in 2000. Therefore, the nanocrystal might an effective way to improve the solubility and bioavailability.

Nanocrystals can be generated by two basic approaches, bottom-up (controlled precipitation/crystallization) and top-down technologies as well as nanonizing (reduction of large size drug particles to smaller ones by mechanical grinding down)^17^. In case of bottom-up technique, organic solvent is used to dissolve the drug and precipitated by adding an anti-solvent in the presence of a stabilizer. As a result, homogenous and smaller size particles are formed^18^. In addition, it may result in the production of amorphous drug nanoparticles which have high dissolution rate as well as saturation solubility^19^. Whereas, top-down technique involves the reduction of particle size by using various processes like micro-fluidization, high pressure homogenization and media milling^20^. The combination techniques, combining a pre-treatment with a subsequent size reduction step are also widely employed for obtaining more homogenous nanoparticles. In this study, the Cyx nanosuspension were prepared by acid–base neutralization and high pressure homogenization, a combination technique of bottom-up and the top–down technologies to improve the solubility. The enhanced solubility, dissolution and pharmacokinetics of Cyx nanosuspension are evaluated.

## Results

### Effect of stabilizer on the stability of nanosuspension

**Figure 1:**
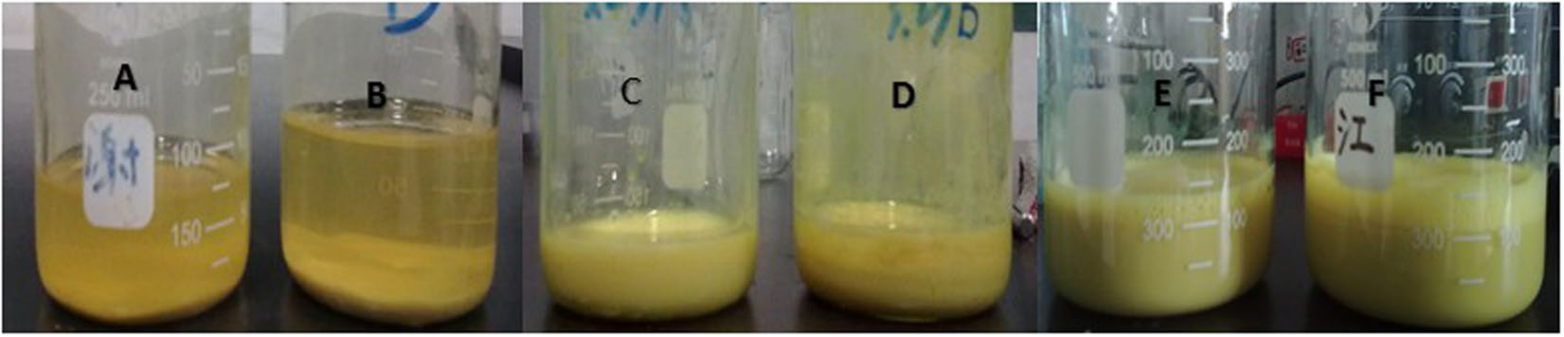
The stability of cyadox nanosuspension prepared with different stabilizer. **(A)** Poloxamer 407; (**B**) Poloxamer 188; (**C**) Sodium alginate; (**D**) Pluronic®F-68; (**E)** Polyvinyl pyrrolidone; (**F)** Polyvinyl alcohol.

As illustrated in Fig. 1, the Cyx nanosuspension stabilized with poloxamer 407 and poloxamer 188 rapidly produced sedimentation after standing for 5 min. When using sodium alginate or Pluronic®F-68, the nanosuspension showed different degrees of agglomerations and poor mobility. The nanosuspension prepared of polyvinyl pyrrolidone and polyvinyl alcohol was uniform yellow suspension and hardly produces sedimentation. When stood for 60d, nanosuspension stabilized with polyvinyl pyrrolidone still did not produce the stratification and sedimentation. These results demonstrated that polyvinyl pyrrolidone might be the best stabilizer.

### Effect of homogenization pressure and cycle times on the sizes of nanosuspension

Under the low homogenization pressure of 300 bar, the sizes of nanosuspension were decreased from 738.9 ± 103.0 to 636.0 ± 49.32 nm when the cycle times was increased from 1 to 3, but showed not obvious change when the cycle times still continued to be increased. As the homogenization pressure was enhanced to 1200 bar, the sizes of nanosuspension was decreased from 524.1 ± 58.12 to 364.3 ± 34.28 nm when the cycle times was increased from 1 to 6 but displayed no significant change as the cycle times was increased up to 10. These results demonstrated the best preparation process was 300 bars for 3 cycles and followed by 1200 bar for 6 cycles (Table 1).

**Table 1:** Effect of homogenization pressure and cycle times on the sizes of nanosuspension.

Homogenization pressure (bar)	Cycle times	Particle sizes (nm)
300	1	738.9 ± 103.0
300	3	636.0 ± 49.32
300	6	645.1 ± 55.14
300	10	652.0 ± 50.27
1200	1	524.1 ± 58.12
1200	3	460.9 ± 29.52
1200	6	364.3 ± 34.28
1200	10	325.7 ± 40.61

### Properties of cyadox nanosuspension

**Figure 2:**
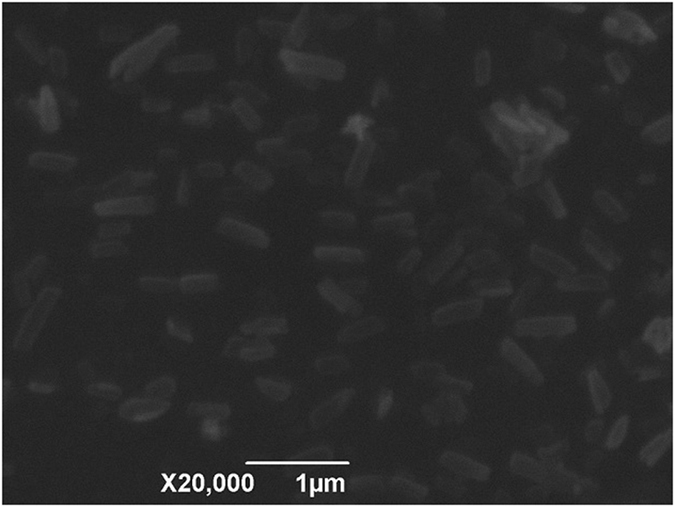
SEM photomicrograph of Cyx nanoparticles (×20,000).

Cyx nanosuspension was a uniform suspension of yellow ivory color. The SEM image revealed that the nanocrystals were long strip in shapes having smooth surface and dispersed well with even particle size distribution without agglomerations (Fig. 2). Moreover, PCS analysis revealed that the mean nanocrystals sizes ranged 389.8 ± 13.1 nm with PDI 0.440 ± 0.028 as well as zeta potential of −32.5 ± 0.4 mV. The Cyx contents, sedimentation rate as well as pH values of nanosuspension were 10%, 1 and 7, respectively. Further, re-dispersion time was only 30 seconds in case of layered suspension under the magnetic shaker rotating at 20 r/min.

### Identification

**Figure 3:**
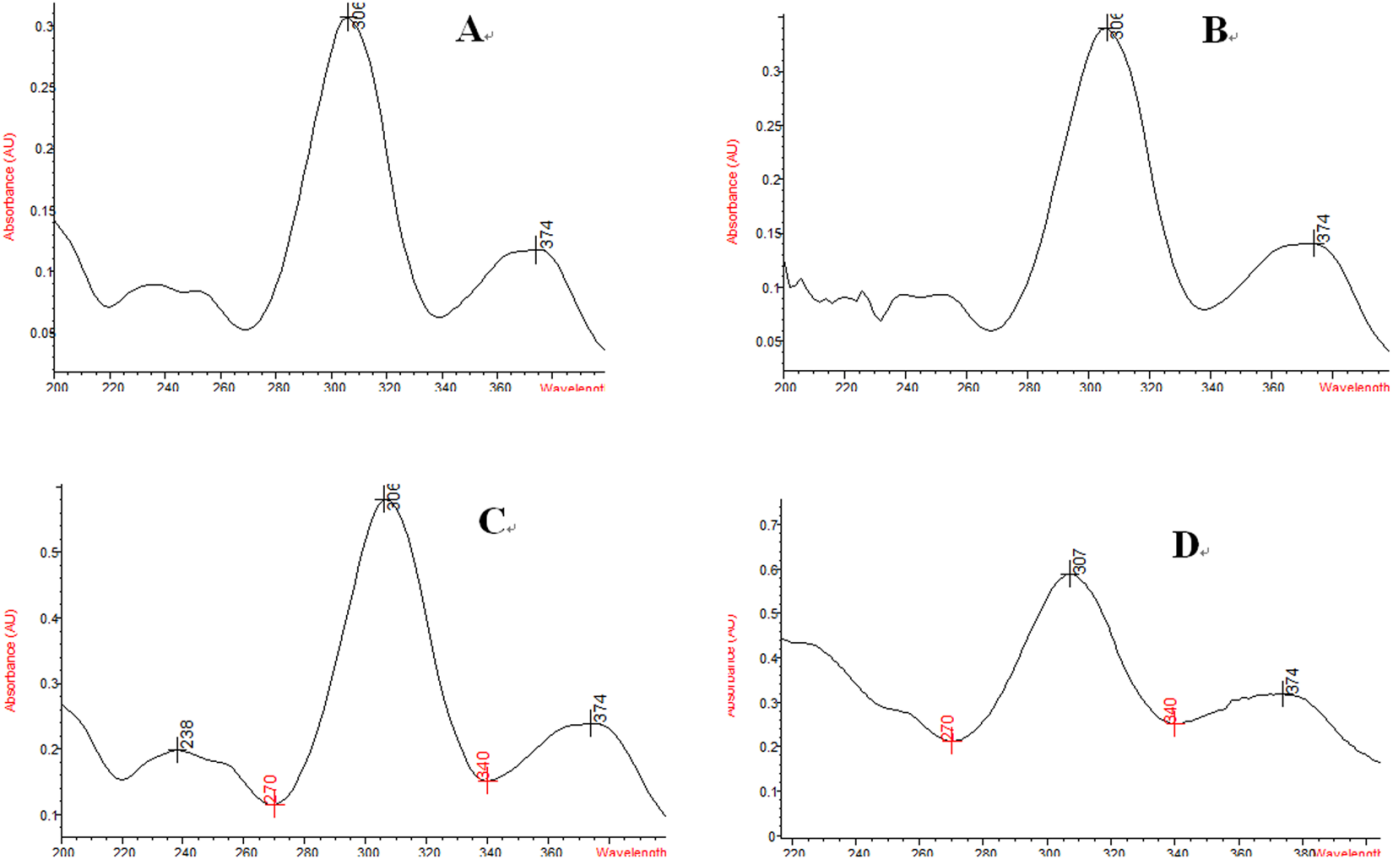
The UV scanning spectrum of Cyx preparations and standards. (**A**) Cyadox standards; (**B~D**) Cyadox preparations from three independent batches.

As showed in Fig. 3, the UV scanning spectrum of extracted Cyx from nanosuspension was consistent with Cyx standard with maximum absorption peak at 306 nm and a second maximum absorption peak at 374 nm.

### Solubility of Cyx nanocrystal

The saturation solubility of Cyx nanocrystal in different solvent including both aqueous solution and organic solvent were enhanced by 1.2~127.7 folds compared to bulk Cyx, respectively (Table 2). The solubility enhancement was most significant in water. The saturation solubility of Cyx nanocrystal was 1415.822 μg/mL in water and 127.7-fold higher than that of bulk Cyx (11.086 μg/mL).

**Table 2:** Solubility of bulk Cyx and Cyx nanocrystal in various solvents (25 °C, standard atmospheric pressure).

Solvents	Solubility (µg/mL, $\overline{{\rm{X}}}$ ± S.D.)		Enhanced folds
	Bulk Cyx	Cyx nanocrystal	
Water	11.086 ± 0.013	1415.822 ± 0.008**	127.7
Ethanol	19.395 ± 0.010	942.496 ± 0.044**	48.6
Methanol	23.889 ± 0.003	526.485 ± 0.082**	22.0
Acetonitrile	65.966 ± 0.139	228.701 ± 0.002**	3.5
Acetone	66.429 ± 0.008	272.712 ± 0.002**	4.1
DMSO	3456.442 ± 0.033	4204.742 ± 0.003**	1.2
DMF	5308.013 ± 0.035	11802.890 ± 0.023**	2.2

*Statistical significances compared with bulk Cyx are p < 0.01.

### *In vitro* release

**Figure 4:**
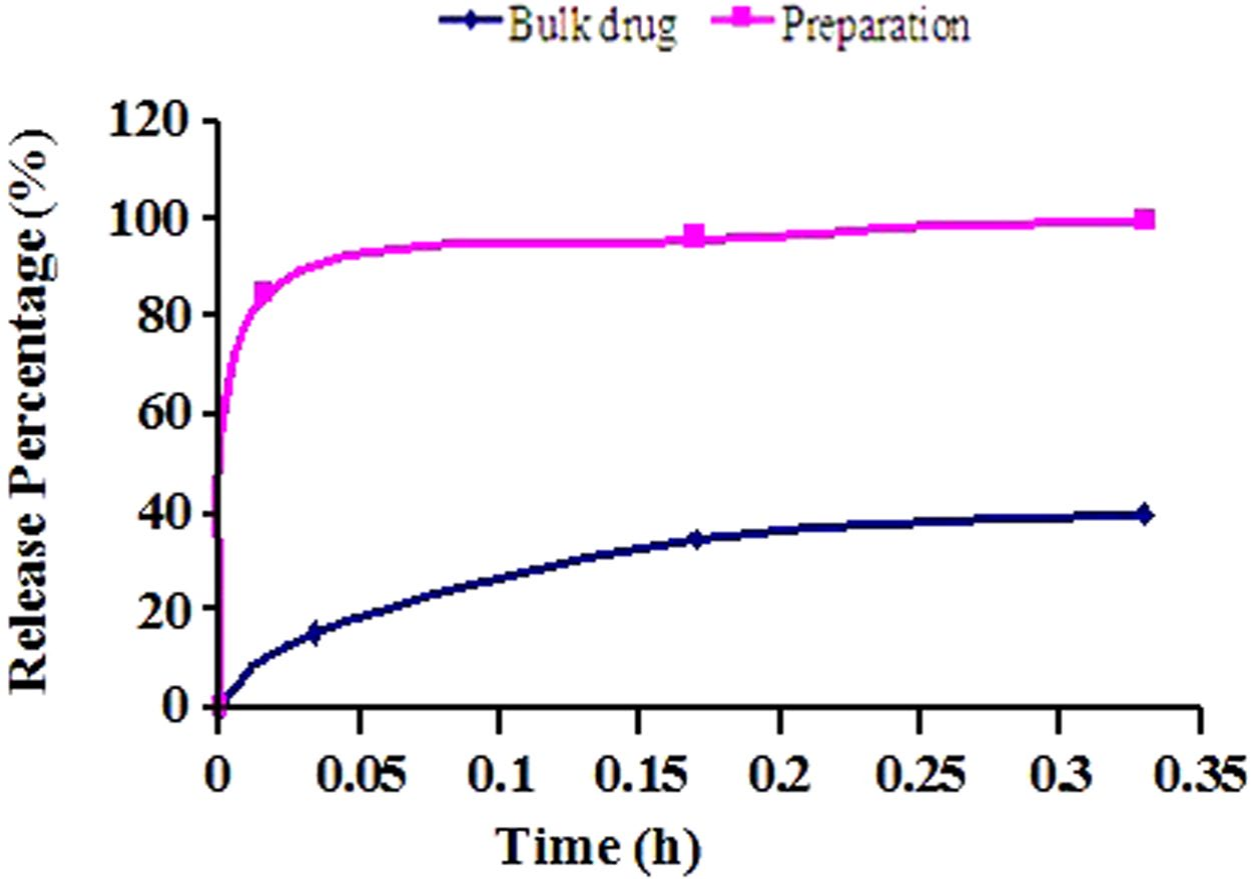
Release profiles of bulk Cyx and Cyx nanosuspension in PBS buffer solution. (n = 6, pH = 7.4).

*In vitro* releases of bulk Cyx and Cyx nanosuspension are illustrated in Fig. 4. The nanosized Cyx displayed an intense increase in rate and extent of release compared with bulk Cyx. Cyx nanosuspension released 99.22% of the total drug within 20 min, while the bulk Cyx only showed 40.02% drug dissolution.

### Pharmacokinetics of Cyx nanosuspension

**Figure 5:**
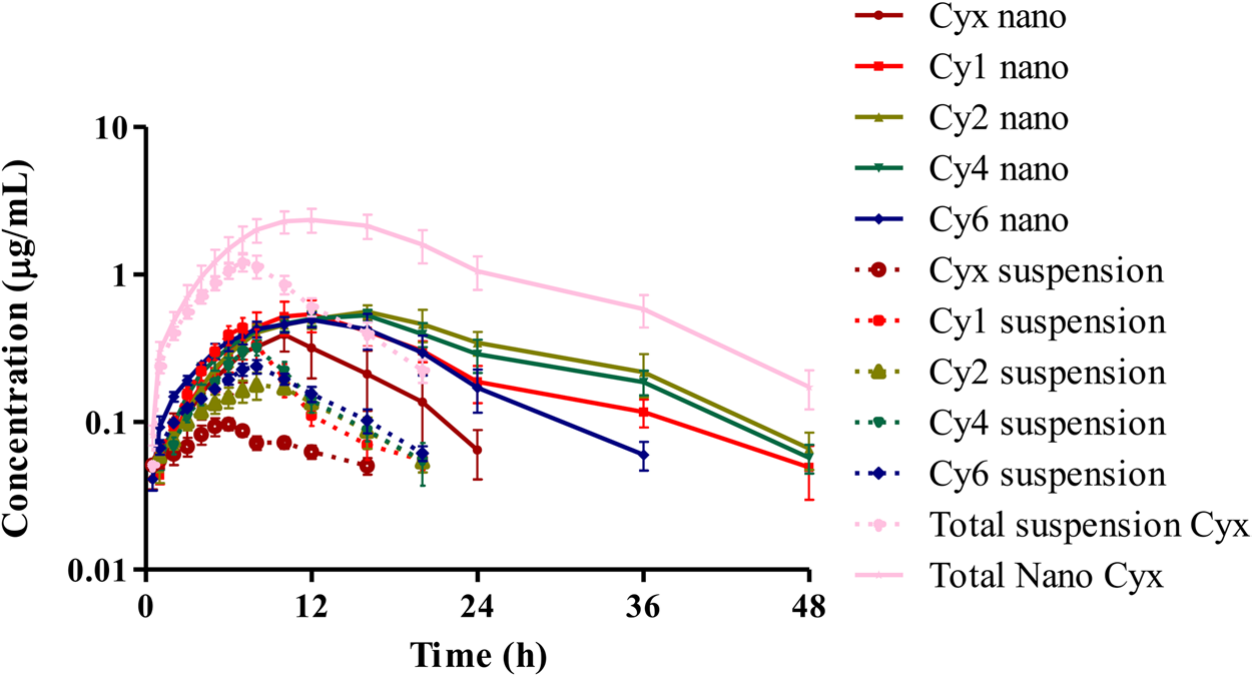
Concentration of prototype and metabolite vs. time curves of Cyx nanosuspension and bulk Cyx after oral administration at dose rate 40 mg/kg b.w. in Beagle dogs.

A pharmacokinetic study of Cyx and its main metabolites was performed in beagle dogs to access the absorption efficiency of Cyx nanosuspension. Plasma concentration-time profiles of Cyx nanosuspension and normal suspension Cyx following oral administration are presented in Fig. 5. After PO delivery, the Cyx nanosuspension and normal Cyx suspension were swiftly reached to its peak concentrations of 0.516 ± 0.07 and 0.22 ± 0.099 µg/mL at 13.67 ± 2.79 and 7.5 ± 1.27 h, then decrease gradually remained in plasma for up to 48 and 20 h, respectively (Fig. 5).

Pharmacokinetic parameters are listed in Table 3. An increase in absorption with nanosuspension was absorbed. The area under concentration time curve (AUC_0-∞_), elimination half-life (T_1/2_), and mean resident time (MRT) of Cyx nanosuspension were 11.24 ± 3.85 h × µg/mL, 9.14 ± 3.03 h and 16.824 ± 3.77 h, respectively, which were superior to Cyx normal suspension delivery with 3.13 ± 1.12 h × µg/mL, 7.51 ± 0.895 h and 9.72 ± 1.67 h, respectively. Moreover, absorption was enhanced compared to normal suspension indicating that the difference in absorption of Cyx is primarily attributed to dissolution and particle size. The bioavailability of Cyx nanoparticles increased up to 359.10%. The nanosuspension of Cyx enhanced the AUC_0-∞_, MRT and T_1/2_ by 3.59, 1.73 and 1.2 folds (Table 3). Thus, nanosuspension avoided the first-pass effect, which is primarily responsible for low bioavailability of Cyx.

**Table 3:** Mean plasma pharmacokinetics of Cyx nano-suspension and normal Cyx in Beagle dogs following oral delivery @ 40 mg/kg.

Parameters	Units	Normal Cyx	Cyx nanoparticles
λz	1/h	0.097 ± 0.012	0.086 ± 0.035
CL	L/h/kg	15.67 ± 9.79	4.46 ± 2.5
T_1/2_	h	7.51 ± 0.895	9.14 ± 3.03
MRT_last_	h	9.72 ± 1.67	16.824 ± 3.77
AUMC _0-∞_	h × h × µg/mL	43.49 ± 17.46	233.1 ± 115.23
T_max_	h	7.5 ± 1.27	13.67 ± 2.79
Vz	L/kg	159.27 ± 75.03	50.35 ± 11.2
AUC_0-∞_	h × µg/mL	3.13 ± 1.12	11.24 ± 3.85
C_max_	µg/mL	0.22 ± 0.099	0.516 ± 0.07
F	%		359.10

λz, elimination rate constant; T_1/2_, elimination half-life; AUMC_0-∞_, area under first moment curve; CL, clearance rate; T_max_, time to reach maximum concentration; C_max_, maximum concentration of drug; AUC_0-∞_, area under plasma concentration-time curve; VZ, volume of distribution; MRT_last_, mean resident time; F, bioavailability. Data are presented as mean ± SD (standard deviation), n = 12.

## Discussion

Although Cyx might be a promising antibacterial agent for infectious diseases in different animal because of its excellent antibacterial activity and safety, there is a big challenge for its clinical application due to its poor solubility in water and different organic solvents. It is reported that the suspension based on the nanocrystal might be an effective formulation for the insoluble drug to improve the solubility, dissolution and bioavailability due to the development the special nature of nanoparticles^21^. Therefore, the cyadox nanocrystal suspension was developed to achieve its clinical application. Since Cyx only has ideal solubility in hot strong basic aqueous but not in neutral water, the drug crystals were prepared by recrystallization based on acid–base neutralization. The drug was firstly dissolved in a hot sodium hydroxide solvent and subsequently precipitated by the swift addition of the same molar quantity of 4 °C hydrochloric acid solvent in the presence of a stabilizer under rapid oscillation. Rapid addition of the 4 °C hydrochloric acid solvent to the hot sodium hydroxide solvent lead to sudden super saturation of Cyx in the neutral water produced by acid-base neutralization reaction, and generation of fine crystalline. As the total surface area of the resulting nanosuspension particles is typically orders of magnitude larger compared to a coarse suspension, large quantities of additives is necessary to ensure adequate stabilization. In this process, the stabilizer is very important to avoid the aggregation of the generated drug crystals. Therefore, a careful evaluation of the type of the stabilizer used is key to the successful production of nanosuspensions^22^. The selection of suitable stabilizer is mainly affected by similar hydrophobicity between the drug and stabilizer^23^. In fact, similar hydrophobicity could consequently resulted in better surface covering and provide better steric stabilization^24^. Besides the surface charge, stabilizer-drug interaction is also an important factor to consider as variable pH condition is affected by electrolytes and it has been proved in earlier findings that ionic stabilization with high zeta potential is not the only essential requisite for nanosuspension stability^25^. In this study, the common used stabilizers for better steric stabilization, such as poloxamer 407, poloxamer 188, sodium alginate, Pluronic®F-68, polyvinyl pyrrolidone and polyvinyl alcohol, were used to achieve the satisfactory^26–29^. The stability of nanosuspension stabilized with polyvinyl pyrrolidone is consistent with our previous study as the suspension had the longest stability without agglomeration and sedimentation using PVP as carrier material^30, 31^.

In order to obtain uniform nanocystal, the high pressure homogenization technique was used by using 2 different ranges of pressure (300–1200 bars) to break the drug crystals to form uniform nanoparticles and to avoid the growth of particles triggered by different saturation solubility in the vicinity of different sized particles, which was described in Ostwald ripening^32^. It is essential to run many processes through homogenizer (homogenization cycles) in order to get a narrow distribution size. For nanosuspension, the homogenization cycles needed are ranged between 10–20, depending on the hardness of a drug^33^. Based on the effect of cycle times of low and high pressure on the nanoparticle size, the low pressure for 6 cycle times and high pressure for 3 cycle times was adopted. The preparation resulted in consistent with Cyx nanocrystal of 389.8 ± 13.1 nm with narrow size distribution and without agglomeration. Moreover, small particles size would be beneficial for better drug absorption and good stability of suspension. The good stability without agglomeration also might be due to the high zeta potential with higher than 30 mV and the steric hindrance of the polymers on the surface of the nanoparticles. The additional assessment presented that the nanosuspension had exceptional sedimentation rate and redispersibility. The pH value of the nanosuspension is 7, suggesting that the nanosuspension has good tolerance in animal. The UV scanning spectrum demonstrated that Cyx in the nanosuspension had the same UV absorption peak as that of standard drug, revealed that preparation process did not show any adverse impact on the structure of drug. These results demonstrated that the combination techniques, combining a recrystallization with a subsequent size reduction step are effective way to obtain the homogenous nanoparticles.

More importantly, the solubility of Cyx in different solvents was significantly increased when formulated as nanosuspensions. The increased saturation solubility was due to increasing the surface area of the nanosized particles. According to Noyes–Whitney equation, an increase in saturation solubility and decrease in particle size leads to an increase dissolution rate^34^. For particle sizes less than 2 μm, the hyperbolic relationship between the size and dissolution rate is significantly pronounced. At a particle size below 1 μm, the dissolution is very fast and completed in a few minutes^35^. Therefore, the nanosuspension released 99.22% of the total drug within 20 min.

The Cyx could be rapidly metabolized and the main metabolites of 1,4-bisdesoxycyadox (Cy1), cyadox-1-monoxide (Cy2), (N-(quinoxaline-2-methyl)-cyanide acetyl hydrozine (Cy4) and quinoxaline-2-carboxylic acid (Cy6) were determined in this study. In order to compare the pharmacokinetic differences, the pharmacokinetics was calculated using the total concentrations of Cyx and its main metabolites at each determined time point. From the Fig. 5, the initial absorption slope for Cyx nanosuspension was not significantly improved compared to normal Cyx suspension when dogs were administered with Cyx dissolved in 0.5% sodium carboxymethyl cellulose orally through gavage were lower as compared to Cyx nanosuspension (Fig. 5). This might be due to that a large intragastric administration of Cyx nanosuspension still exists as nanoparticles. The total dissolved amount in stomach (Gastric fluid volume below 20 mL) of beagle dogs were below 28.31 mg calculated by the 1415.822 μg/mL saturation solubility of Cyx nanocrystal in water, while the orally administered drug for 10–12 kg beagle dogs at dose of 40 mg/kg were 400–480 mg. Therefore, almost nine out of ten administrated drugs still exists in the form of nanoparticles. The nanosuspension significantly prolonged drug absorption, systemic circulation time and improved the bioavailability compared to traditional suspension after oral delivery through gavage (40 mg/kg).

The improved bioavailability and drug absorption due to Cyx nanosuspension might be due to the reduction of particle size and increased saturation solubility. Owing to their small particle size, nanoparticles may exhibit increased adhesiveness to the gastrointestinal tract wall or entered in the intervillar spaces, thus increasing their residence time in the gastrointestinal tract and absorption. In addition, some nanoparticles do not have direct access to the bloodstream. Instead, they are taken up by intestinal lymph vessels and lymph nodes and thus lead to extended drug absorption and longer T_max_. In earlier studies, significant lower bioavailability was observed following oral administration of Cyx^36, 37^. In dogs, the oral bioavailability was only 4.75% while in pigs, the bioavailability was 2.75%^36, 37^. But here, nanosuspension improved the bioavailability (359.10%) which could be beneficial for intestinal bacterial infection in animals. Further, C_max_ (0.516 ± 0.07 µg/mL) and AUC_0-∞_ (11.24 ± 3.85 h × µg/mL) were increased by 2.3 and 3.6 folds, respectively with the particles size reduction which might give rise to pronounce fluctuations in plasma concentration and more intensified antibacterial effects. The terminal half-life and mean resident time of Cyx nanosuspension had also increased 1.2 and 1.73 folds, respectively, which showed that nanosuspension can stay inside the body for longer time. *In vivo* pharmacokinetics in beagle dogs revealed that nanosuspension could improve the bioavailability of Cyx and plasma concentrations were more stable and remained in plasma for longer time than that of conventional suspension. It was a promising approach to improve the oral bioavailability, minimizing the frequency of drug administration and increase the retention time the poorly soluble drugs.

In summary, Cyx nanosuspension was successfully prepared by recrystallization based on acid–base neutralization coupled with high pressure homogenization technique and by using PVP as stabilizer. The nanosuspension had uniform particle distribution, redispersibility as well as exceptional sedimentation rate. The nanosuspension significantly improved the solubility, dissolution, and bioavailability of Cyx in beagle dog. The nanosuspension could be an efficient approach to resolve the challenge of Cyx formulation development.

## Materials and Methods

### Chemicals

Cyx (C_12_H_9_N_5_O_3_, CAS NO. 65884-46-0, molecular weight 271.23 g/mol, 98% purity) and four of its major metabolites including 1,4-bidesoxycyadox (Cy1), cyadox-1-monoxide (Cy2), N-(quinoxaline-2-methyl)-cyanide acetyl hydrazine (Cy4) and quinoxaline-2-carboxylic acid (Cy6) were the product of Institute of Veterinary Pharmaceuticals, Huazhong Agricultural University (Wuhan, China). Other chemicals such as polyvinylpyrrolidone K30 (PVP), hydrochloric acid (HCL), methanol (MeOH), dimethyl sulfoxide (DMSO), sodium hydroxide (NaOH), potassium dihydrogen phosphate (KH_2_PO_4_), sodium chloride (NaCl), ethanol (C_2_H_5_OH), potassium chloride (KCl), disodium hydrogen phosphate dodecahydrate (Na_2_HPO_4_.12H_2_O), carbon tetrachloride (CCl_4_) N, N-dimethylformamide (DMF), acetonitrile (CH_3_CN), and acetone (CH_3_COCH_3_) were commercially obtained from Sinopharm Chemical Reagent Co., Ltd.(Shanghai, China). Deionized water, used for all experiments, was purified using Millipore^®^ purified water system (Milli-Q Co., Ltd, France). All other chemicals and reagents used in this experiment were of highly analytical grade.

### Animal

A total of 12 healthy beagle dogs (6 males, 6 females), 5–6 month of age and uniform weights (10–12 kg) were obtained from Animal Experimental Center of Tongji University, Wuhan, China. The animals were kept under standard condition of temperature and relative humidity ranged between 22 ± 2 °C and 45–65%, respectively. The dogs were offered 400 g of certified commercial diet (Ke Ao Xie Li Co., Beijing, China) at fixed time every day and water was available around the clock throughout the experiment. Each dog was kept in an isolated steel cage (1000 × 10000 × 900 mm) with a precise ear tag number 2 weeks prior to start of experiment for acclimatization. All dogs enjoyed outside walk twice in a week. Experiments were performed in accordance with NIH publication 85–23 “Guide for the Care and Use of Laboratory Animals”^38^ and approved by the Ethical Committee of the Faculty of Veterinary Medicine at Huazhong Agricultural University (HZAUDO-2016-005, 2016-10-26).

### Preparation of cyadox nanosuspension

Cyx nanosuspension was produced by recrystallization based on acid–base neutralization combined high pressure homogenization. Briefly, 10 g Cyx was dissolved in 85 mL hot mixed solution containing 15 mmol NaOH and 2 g stabilizers (poloxamer 407, poloxamer 188, sodium alginate, Pluronic®F-68, polyvinyl pyrrolidone or polyvinyl alcohol). After the dissolution of drug, 15 mL 4 °C 1.0 mol/L HCL solution was quickly poured into the mixed solution under stirring by using a thermostat magnetic stirrer (90–1, Shanghai HuXi analysis instrument factory Co., Ltd., Shanghai, China) to recrystallize the Cyx based on acid–base neutralization. The recrystallized Cyx passed through a high-pressure homogenizer (APV-2000, APV Co., Ltd, Unna, Germany) for 3 cycles at 300 bar and finally 6 cycles at 1200 bar to obtain uniform small particle size. The nanosuspensions were collected into glass vials, labeled, and used for subsequent tests.

### Scanning electron microscopy

The nanoparticles morphology was measured by scanning electron microscopy (SEM) (JSM-6360LV, JEOL Inc., Japan). A very small drop of sample was poured on the glass slide and then quickly oven dried. Followed, the samples were fixed on SEM stub by using double sided adhesive tape and coated with gold at 20 mA for 2 mins by using an auto-fine coater (Ion sputter JFC 1600). Following coating, digital images of samples were attained by SEM with secondary electron detector having an accelerating voltage of 15 kV.

### Determination of size, polydispersity index and Zeta potential

The size and polydispersity index (PDI) of Cyx suspension was evaluated by Malvern Zetasizer ZS3600 (Malvern Instrument, Malvern, UK) at 25 °C at a scattering angle of 90° after suitable dilution. Zeta potential of nanosuspension was measured by using disposable Zeta cells for Zeta potential analysis using electrophoretic mobility technique (Zetasizer ZS3600, Malvern Instrument, Malvern, UK).

### Identification

The structure of Cyx in the nanosuspension was evaluated by using UV spectrophotometer. Drugs extracted using acetonitrile-water (10:90) from 3 bathes of nanosuspensions were diluted up to 4 µg/mL, followed scanned through UV spectrophotometer at a wavelength ranged between 200–400 nm for comparison of UV spectrum with the equal concentration of Cyx standard solution.

### Sedimentation rate

In order to determine the sedimentation rate, the original height (H_0_) of 50 mL nanosuspension was recorded after shaking in a 100 mL graduated cylinder. The height (H) of sedimentation was measured in graduated cylinder after 3 h of standing and sedimentation rate was calculated according to following equation:

$${\rm{F}}={\rm{H}}/{{\rm{H}}}_{0}.$$

### Redispersibility

50 mL of nanosuspension was stood in a 100 mL graduated amber laboratory bottle for three month to produce nanoparticle settling, followed by shaking of layered nanosuspension under magnetic shaker rotating at 20 r/min to calculate its redisperse time.

### pH value

The pH values of suspension were examined with the help of pH indicator paper (Shanghai Sss Reagent Co., Ltd, China) according to the instruction.

### Determination of solubility

Nanosuspension was lyophilized for 48 h (Freeze Dry System; Labconco, America) to determine the saturation solubility. An excess amount of lyophilized Cyx nanocrystals and standard were added into 5 mL 25 ± °C water, ethanol (C_2_H_5_OH), methanol (CH_3_OH), N, N-dimethylformamide (DMF), acetonitrile (CH_3_CN), and acetone (CH_3_COCH_3_), respectively. The different solution after shaken for 30 min at 25 °C was centrifuged at 15000 × g for 10 min and then Cyx in the supernatant was analyzed by high-performance liquid chromatography (HPLC).

### *In vitro* release

*In vitro* release of cyadox nanosuspension was studied in pH 7.4 PBS (every liter contained 0.2 g KCl, 0.2 g KH_2_PO_4_, 8.0 g NaCl and 2.9 g Na_2_HPO_4_·12H_2_O) by using a paddle type dissolution apparatus (Dissolution Tester, RC806, Tianjin Tianda Tianfa science and technology Co., Ltd., Tianjin, China) following guideline of “Peoples’ Republic of Veterinary Pharmacopoeia”. Cyx nanosuspension (0.3 mL, 10 g:100 mL) and bulk 30 mg Cyx were added into six different dissolution cup contained 900 mL degassed pH 7.4 PBS preheat at 37 ± 0.5 °C under Kaplan stirring at 50 rpm, respectively. 1 mL of sample taken from receiver solution at fixed time points, while same volume of PBS was taken to keep the persistent volume. After filtration, the Cyx contents in samples were measured through HPLC.

### Pharmacokinetics study

Evenly sexed twelve clinically healthy and acclimatization beagle dogs of 5–6 months of age with uniform weights (10–12 kg) were used to evaluate the pharmacokinetics of Cyx nanosuspension and bulk Cyx suspended into 0.5% sodium carboxymethyl cellulose aqueous solution. The dogs were randomly divided into 2 groups with even sex and 6 dogs were in each group before the start of experiment. Each group was randomly orally administered by the Cyx nanosuspension and bulk Cyx at the dose rate of 40 mg/kg, respectively. At different time points of 0.25, 0.50, 1, 2, 3, 4, 5, 6, 7, 8, 10, 12, 16, 20, 24, 36, 48 and 60 h of administration, blood samples (1 mL) were taken from cephalic vein into heparin-containing tubes. The samples were prepared according to the method described in earlier study by our research team for detection of Cyx and its metabolites in plasma using HPLC system^37^. The data on plasma drug concentration-time were analyzed using non-compartmental pharmacokinetics by using Winnonlin software (Version 5.2.1, Pharsight Co. CA, USA) according to earlier reports^39, 40^.

### HPLC assay

All the samples were analyzed using HPLC (Waters 2695 series) combined with an ultraviolet detector system with the wavelength set at 320 nm. For *in vitro* study, the chromatographic separation was achieved with an analytical ZORBAX SB C_18_ column (250 × 4.6 mm, i.d. 5 µm; Agilent Technology, USA) at 30 °C. The mobile phase was acetonitrile and Milli-Q water with the proportion of 20/80 (V/V). The flow rate and injection volume were 0.8 mL/min and 10 μL, respectively. Linearity of the standard curve was ranged from 0.1 to 20 µg/mL and the correlation coefficient (r) values were 0.9993. The LOD and LOQ were 0.05 µg/mL and 0.1 µg/mL, respectively. Inter- and intraday relative standard deviations were <3.0%.

For all plasma samples, an aliquot of 0.3 mL of plasma from each time point was added into 1.5 mL polypropylene centrifuge tube. Deproteinization was done with 0.3 mL methanol followed by vortexing and centrifugation (10000 × g for 10 min at 4 °C). Afterwards, supernatant was filtered through 0.22 µm microbore cellulose membrane and 40 µL of each sample was injected onto the HPLC column. Separation of chromatographs was achieved using a ZORBAX SB-C 18 column (250 mm × 4.6 mm i.d., 5 µm, Agilent, USA) at a flow rate of 1 mL/min at 30 °C. The gradient elution mode was set for separation of Cyx and its main metabolites, with the mobile phase A containing 0.5% formic acid and mobile phase B containing pure acetonitrile. The gradient profile was set as follows: 0 min, 88% A; 5 min, 80% A; 26 min, 72% A; 26.01 min, 88% A; 30 min, 88% A. Linearity of the standard mixtures with different concentrations in plasma (0.02 to 10.24 µg/mL) showed excellent results within the same day as well as on different days. The correlation coefficient (r) values of all five metabolites indicating functional linear relationships at different concentrations were >0.999 across the concentration ranges used for all samples. The LOD was 0.02 µg/mL for Cyx, Cy1, Cy2 and Cy6 and 0.04 µg/mL for Cy4. LOQ in dog plasma was 0.05 µg/mL for all analytes, except Cy4, for which an LOQ of 0.08 µg/mL was obtained. The mean recovery rate was >80% in plasma. Inter- and intraday relative standard deviations were <8%, signifying that the proposed method is accurate and precise for detection of Cyx and its major metabolites in plasma.

### Statistical analysis

The data were analyzed using one-way analysis of variance (ANOVA) by SPSS 11.0 programme for window (SPSS Co. USA). The significant difference and extremely significant difference were determined as p-value of 0.05 and 0.01, respectively.

### Ethic Statement

The use of dogs in this study was according to Animal Experimental Ethical Inspection of Laboratory Animal Center, Huazhong Agricultural University, Wuhan, China (HZAUDO-2016-005, 2016-10-26). All efforts were made to minimize the suffering of the animals.
